# Long-Range Control of *Renin* Gene Expression in Tsukuba Hypertensive Mice

**DOI:** 10.1371/journal.pone.0166974

**Published:** 2016-11-18

**Authors:** Aki Ushiki, Hitomi Matsuzaki, Junji Ishida, Akiyoshi Fukamizu, Keiji Tanimoto

**Affiliations:** 1 Graduate School of Life and Environmental Sciences, University of Tsukuba, Tsukuba, Ibaraki, Japan; 2 Faculty of Life and Environmental Sciences, University of Tsukuba, Tsukuba, Ibaraki, Japan; 3 Life Science Center, Tsukuba Advanced Research Alliance (TARA), University of Tsukuba, Tsukuba, Ibaraki, Japan; Max Delbruck Centrum fur Molekulare Medizin Berlin Buch, GERMANY

## Abstract

Renin, a rate-limiting enzyme in the renin–angiotensin system, is regulated to maintain blood pressure homeostasis: *renin* gene expression in the kidney is suppressed in a hypertensive environment. We found that expression of a 15-kb human *RENIN* (h*REN)* transgene was aberrantly upregulated (>4.2-fold), while the endogenous mouse *renin* (m*Ren*) gene was suppressed (>1.7-fold) in Tsukuba hypertensive mice (THM), a model for genetically induced hypertension. We then generated transgenic mice using a 13-kb m*Ren* gene fragment that was homologous to the 15-kb h*REN* transgene and found that its expression was also upregulated (>3.1-fold) in THM, suggesting that putative silencing elements of the *renin* genes were distally located in the loci. We next examined the possible role of a previously identified mouse distal enhancer (mdE) located outside of the 13-kb m*Ren* gene fragment. Deletion of the mdE in the context of a 156-kb m*Ren* transgene did not affect its transcriptional repression in THM, implying that although the silencing element of the m*Ren* gene is located within the 156-kb fragment tested, it is distinct from the mdE. Consistent with these results, deletion of the 63-kb region upstream of the mdE from the endogenous m*Ren* gene locus abrogated its transcriptional repression in THM. We finally tested whether dysregulation of the short *renin* transgenes also occurred in the fetal or neonatal kidneys of THM and found that their expression was not aberrantly upregulated, demonstrating that aberrant regulation of short *renin* transgenes commences sometime between neonate and adult periods.

## Introduction

The renin–angiotensin system is a vasopressor signaling cascade that plays a pivotal role in blood pressure regulation and electrolyte homeostasis. The first and rate-limiting reaction in this cascade is catalyzed by renin, which is predominantly synthesized in the juxtaglomerular (JG) cells of the kidney. Renin cleaves its unique substrate, angiotensinogen (AGT), to generate angiotensin I. By the action of the angiotensin-converting enzyme (ACE), angiotensin I is further converted to angiotensin II, which increases blood pressure through vasoconstriction and aldosterone secretion by binding to angiotensin receptors. Because renin activity is reflected in blood pressure changes, renin expression is strictly regulated through a feedback mechanism initiated by various physiological stimuli to maintain blood pressure homeostasis [1]. For example, *renin* gene transcription is activated and suppressed in hypertensive and hypotensive environments, respectively, *in vivo* [2–4].

JG cells are located in the walls of the afferent arterioles of the glomeruli, and because of this unique spatial configuration, *renin* genes can sense blood pressure changes through renal baroreceptors, *macula densa* signals, renal sympathetic input, and angiotensin II receptor-mediated feedback signaling [1, 5]. Through cell-surface G protein-coupled receptors, these extracellular stimuli are converted into changes in cellular cAMP levels, which then play a pivotal role in the regulation of *renin* gene transcription [6, 7]. Although physiological stimuli leading to altered *renin* gene transcription have been intensively investigated [8–10], *cis*-DNA sequences and *trans*-factors involved in these processes are not well understood.

To date, while numerous proximally located *cis*-regulatory elements of *renin* have been identified using cultured cells (such as immortalized mouse renin-producing renal tumor As4.1 cells [11, 12]), their *in vitro* and *in vivo* significance sometimes appears to differ [13, 14], and such discrepancies may reflect the difficulty in reconstituting a complex physiological microenvironment in a culture dish. In addition, because of the size limitation of DNA fragments that can be introduced into plasmid vectors for reporter assays, it is likely that only a fraction of *cis*-regulatory elements, primarily those located close to the coding region, have been preferentially identified.

Recent advances in genome-wide analysis have revealed that gene transcription is frequently regulated by distal regulatory elements through looping interactions with target promoters [15]. Examination of transgenic mice (TgM) carrying human *RENIN* (h*REN*) gene fragments of various sizes suggested the existence of long-range regulation in *renin* gene transcription [16, 17]. Sigmund and colleagues generated TgM using 140-kb or 13-kb h*REN* gene fragments, carrying 5'-flanking sequences of 35 kb or 900 bp, respectively, and tested their angiotensin II-induced hypertension responsiveness [3, 4, 17]. They found that although h*REN* gene expression from the 140-kb transgene (Tg) as well as the endogenous mouse *renin* (m*Ren*) gene was appropriately suppressed in their hypertension model animals, the 13-kb h*REN* Tg expression was inappropriately upregulated. This suggested that a silencer element for the h*REN* gene is located outside of the 13-kb fragment but within the boundaries of the 140-kb Tg. However, because only one Tg line was investigated in this analysis, it may have been subjected to position-of-integration site effects thereby generating aberrant regulation. It is also possible that the result was the mere reflection of a species-specific difference in regulation of the two homologues.

In the m*Ren* and h*REN* genes, distal enhancer elements have been identified 3 and 12 kb upstream, respectively, of their transcription start sites in *in vitro* experiments [18–20]. The mouse distal enhancer (mdE) is comprised of multiple binding sites for transcription factors, including a cyclic AMP-responsive element (CRE) [18, 20]. Using transgenic and knockout mice, we and others have shown that these enhancers are essential for basal transcriptional activity of the genes [21, 22]. Although its essential role in full induction of the m*Ren* gene transcription in ACE inhibitor-induced hypotensive status has been established [22, 23], its role in a hypertensive environment has not been determined.

**Fig 1:**
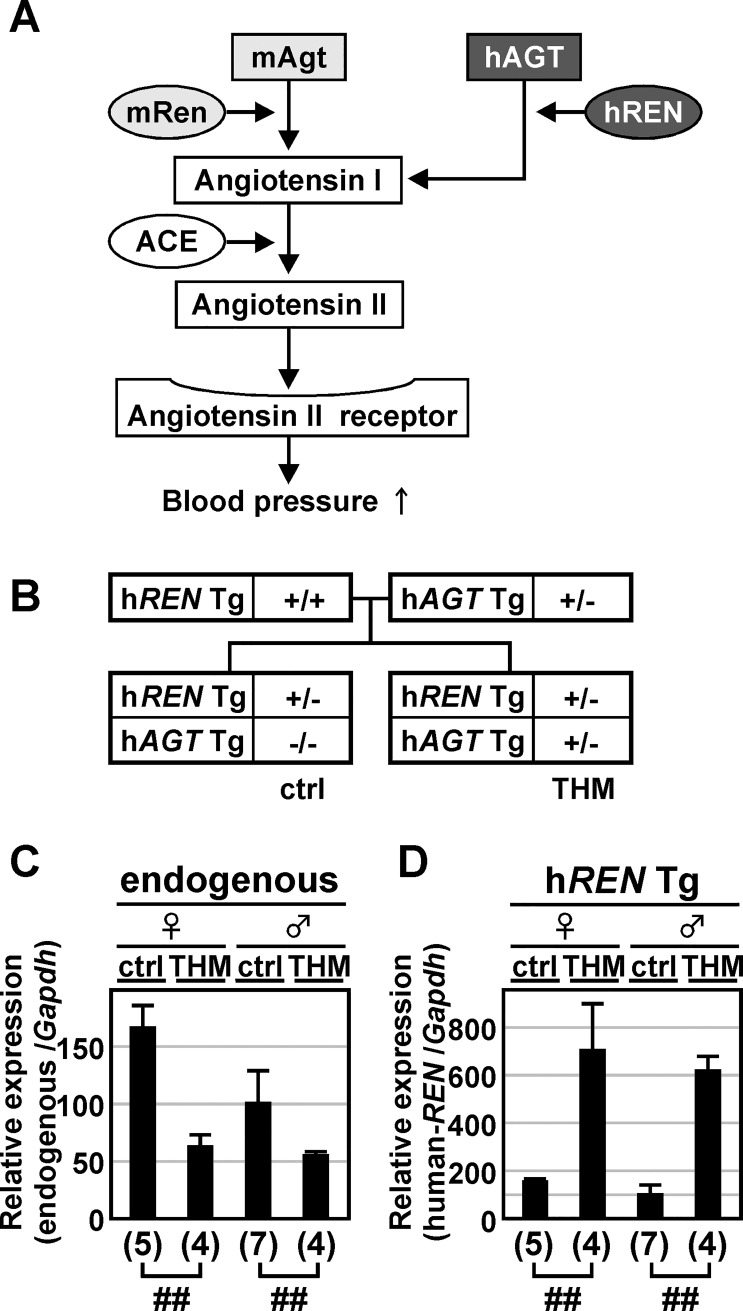
Expression of endogenous mouse *Renin* and human *RENIN* transgenes in THM. **(A)** Schematic representation of chimeric renin-angiotensin system in Tsukuba hypertensive mice. mRen, mouse Renin; mAgt, mouse Angiotensinogen; hREN, human RENIN; hAGT, human ANGIOTENSINOGEN; ACE, Angiotensin-converting enzyme. **(B)** Breeding strategy for obtaining normotensive (control; ctrl) and hypertensive mice (Tsukuba hypertensive mice; THM). h*REN*, human *RENIN*; h*AGT*, human *ANGIOTENSINOGEN*; Tg, Transgene. **(C** and **D)** Total RNA was isolated from the kidney of normotensive (ctrl) or hypertensive (THM) TgM (8-week old). Levels of endogenous mouse *Ren* (endogenous; C) or human transgenic *REN* (h*REN* Tg; D) gene expression were analyzed by qRT-PCR. Each value represents the ratio of endogenous m*Ren* or h*REN* Tg gene expression to that of *Gapdh*. The expression value of male control animals in each group was arbitrarily set at 100. qPCR analyses were repeated three times. Number of animals analyzed is shown in parentheses below each panel and mean ± SD are shown. Statistically significant differences between the control animals and THM were determined using an unpaired t-test (##, *P* < 0.01).

We previously generated a mouse model of angiotensin II-induced hypertension (the Tsukuba hypertensive mouse; THM) that overexpresses both a 15-kb h*REN* Tg bearing 2.8-kb of 5'-flanking sequence and a 14-kb h*AGT* Tg (Fig 1A, [24]). Because of strict species specificity for enzymatic reaction in renin–angiotensin system, hREN and mRen selectively catalyze hAGT and mAgt, respectively, to generate angiotensin I [25]. Therefore, a TgM carrying h*REN* alone exhibits normal blood pressure (systolic blood pressure [SBP], 97.0 ± 7.3 mmHg; [24]). However, because the amino acid sequences of angiotensin I are identical in human and mouse, TgM carrying both h*REN* and h*AGT* genes reproducibly exhibit chronic hypertension, at least in adults (SBP, 129.1 ± 7.1 mmHg; [24]), as a result of overproduction of angiotensin II by murine ACE. On the other hand, because *renin* gene transcription is strictly regulated to maintain blood pressure homeostasis, it is quite intriguing that THM exhibits severe hypertension.

We therefore hypothesized that the 15-kb h*REN* Tg in our disease model might be also subject to aberrant regulation, as was the case for the 13-kb h*REN* Tg [3, 4, 17], and that distal regulatory elements of both human and mouse *renin* genes might be essential for their transcriptional repression in THM. To test this hypothesis, we generated TgM carrying the 13-kb m*Ren* gene fragment that was homologous to the 15-kb h*REN* Tg, as well as m*Ren* knock-out mice carrying a 63-kb deletion in its 5' upstream region, and these and other mutant *renin* gene alleles were subjected to the THM environment to examine their transcriptional responses *in vivo*. These studies demonstrated that distal silencer element of the m*Ren* gene was essential for its proper regulation in adult, but not in fetal and neonatal THM.

## Materials and Methods

### Construction of the m*Ren* gene fragment and generation of TgM

A BAC clone carrying the mouse *Ren-1c* gene (RPCI23-240p23, GenBank accession no. AC068906) was obtained as described elsewhere [14]. To facilitate long-range structural analysis of the Tg and discriminate the transcripts of transgenic from endogenous m*Ren* loci, *Sfi*I and *Pvu*II restriction sites as well as flip recombinase recognition target (FRT) sequences, as a tag were introduced into the 3'-untranslated region of the gene (RPCI23-240P23+FRT, [14]). The transcriptional start site of the *Ren-1c* gene [26] corresponded to the nucleotide (nt) position 89,006 of this clone or chr1:135,247,251 (NCBI37/mm9), and all of the nt positions hereafter are expressed relative to this site (designated as +1).

The 13-kb m*Ren* gene fragment was subcloned from this BAC clone using a prophage-recombination system [27]. To facilitate retrieving vector construction, the following oligonucleotides were annealed (generating *Kpn*I-*Sfi*I-*Spe*I-*Xho*I-*Mlu*I-*Afl*II-*Sfi*I-*Bss*HII-*Sac*I sites) and replaced with the *Kpn*I-*Sac*I portion of the pBluescript II KS (+) to generate pBS-MCS:

5'-CGGCCAAAAAGGCCACTAGTCTCGAGAACGACGCGTCGAACTTAAGGCCAAAAAGGCCGCGCGCGAGCT-3' and 5'-CGCGCGCGGCCTTTTTGGCCTTAAGTTCGACGCGTCGTTCTCGAGACTAGTGGCCTTTTTGGCCGGTAC-3'.

The *Mlu*I-*Xho*I region (nucleotides 94,530–105,069) of the BAC was subcloned into the *Mlu*I/*Xho*I sites of pBS-MCS. The plasmid was then treated with *Afe*I/*Xho*I, blunt-ended, and self-ligated to obtain pmRen-3'fr, which carries a 3'-homology sequence (*Mlu*I [94,530]-*Afe*I [100,425]). The 5'-homology sequence was PCR-amplified using the following primer set and the BAC DNA as a template: 5'-AAAGGCGCGCCGAGGTTAGACTCGAGGTTACTTTTCCA-3' and 5'-TTTCGACGCGTCCAAGCTAGGTAGGTATAGGATAAGCA-3' (*Bss*HII and *Mlu*I sites underlined).

Following *Mlu*I/*Bss*HII digestion, the fragment was cloned into the *Mlu*I site of pmRen-3' fr to derive the retrieving vector. The plasmid was linearized with *Blp*I/*Mlu*I and used to transform *Escherichia coli* cells (strain EL250; a gift from N.A. Jenkins, National Cancer Institute, Frederick, Maryland, USA) harboring the 240P23+FRT BAC [14]. After selection on the basis of ampicillin resistance, transformants that underwent accurate recombination were identified by restriction enzyme digestion and DNA sequencing.

The 13-kb m*Ren* gene fragment was released from the retrieving plasmid by digestion with *Bss*HII, gel purified, and microinjected into the pronuclei of fertilized eggs of ICR mice (Charles River Laboratories Japan, Kanagawa, Japan). Tail DNA from founder offspring was screened first by PCR and then by Southern blotting.

For structural analysis of Tgs, agarose-embedded thymus DNA was treated with *Sfi*I and fractionated by pulsed-field gel electrophoresis. For copy number analysis, the DNA was digested with *Pvu*II and fractionated by conventional agarose gel electrophoresis. Following capillary transfer onto nylon membranes (PerkinElmer, Waltham, MA), blots were hybridized with a [α-^32^P]-labeled DNA probe (nt 98,147–98,713 [AC068906]). Tg copy numbers were determined by comparison with a standard curve generated by spiking non-Tg mouse genomic DNA with varying amounts of plasmids containing the m*Ren* gene sequence. Signal intensities of the bands were quantified by phosphorimager and ImageQuant software (GE Healthcare, Princeton, NJ).

### Generation of mutant alleles by CRISPR/Cas9 genome editing

The following pairs of oligos were annealed, phosphorylated and ligated to *Bbs*I site of pX330 (a gift from Feng Zhang; Addgene plasmid # 42230, [28]) for generating hCas9/gRNA expression vectors. 5'-large-del allele: 5'-CACCGATAGAATGCAGCTCATGTCT-3'/5'-AAACAGACATGAGCTGCATTCTATC -3' and 5'- CACCGAGGGAGAAATAAAGTAGGTG-3'/5'-AAACCACCTACTTTATTTCTCCCTC -3'. pseudo-WT allele: 5'-CACCGCTTGGCCTAGGGTTACTGGG-3'/5'-AAACCCCAGTAACCCTAGGCCAAGC-3'. The CRISPR/Cas9 plasmid (and the donor DNA fragment described below in case for generating pseudo-WT allele) was microinjected into the pronuclei of fertilized eggs of C57BL/6J mice (Charles River Laboratories Japan, Kanagawa, Japan). The donor DNA fragment was PCR amplified by using following primer set and the plasmid bearing 13-kb m*Ren* and the FRT in its 3'-untranslated region as a template: 5'-GGCTGGGATTTAGGATAGG-3'/5'-ACTTTACTAACAAACCTCCATCTC-3'. Tail DNA from founder offspring was screened first by PCR and then by Southern blotting.

For structural analysis of mutant alleles, tail (5'-large-del) or thymus (pseudo-WT) DNA was treated with *Eco*RV and *Pvu*II, respectively, and fractionated by conventional agarose gel electrophoreses. Following capillary transfer onto nylon membranes, blots were hybridized with [α-^32^P]-labeled DNA probes corresponding to nt 86,373–86,793 and nt 98,147–98,713 sequences, respectively [AC068906].

### Animal procedures

Mice were housed in a pathogen-free barrier facility in a 12-hour light/12-hour dark cycle, and fed standard rodent chow. Adult (8-week old) and neonatal (1-day old) mice were sacrificed by cervical dislocation or beheading, respectively, and the kidneys were immediately removed and flash-frozen in liquid nitrogen. For fetal studies, female mice were sacrificed at 17.5 days of gestation (detection of the vaginal plug was designated day 0.5), and fetuses were removed from the uterine sacs. Fetal kidneys were immediately flash-frozen in liquid nitrogen.

Animal experiments were performed in a humane manner under approval from the Institutional Animal Experiment Committee of the University of Tsukuba. Experiments were performed in accordance with the Regulation of Animal Experiments of the University of Tsukuba and the Fundamental Guidelines for Proper Conduct of Animal Experiments and Related Activities in Academic Research Institutions under the jurisdiction of the Ministry of Education, Culture, Sports, Science and Technology of Japan.

### Northern blot analysis

Total RNA was isolated from mouse kidneys using ISOGEN (Nippon Gene, Tokyo, Japan) and analyzed as described elsewhere [14]. A DNA fragment (*Kpn*I-*Nco*I), corresponding to exons 3 to 9 of the *Ren-1c* cDNA (nt 303–1,123; GenBank accession no. NM031192) was [α-^32^P]-labeled and used as a probe. Mouse *Gapdh* gene expression analyzed by a mouse cDNA (nt 565–1,017; GenBank accession no. M32599) probe was used as the internal control.

### qRT-PCR

Total RNA from kidneys was converted to cDNA using ReverTra Ace qPCR RT Master Mix with gDNA Remover (Toyobo, Osaka, Japan). Quantitative amplification of cDNA was performed with the Thermal Cycler Dice (TaKaRa Bio, Shiga, Japan) using SYBR Premix EX Taq II (TaKaRa Bio). PCR primer sequences are as follows: endogenous mouse *renin* gene (5'-GCCCTCTGCCACCCAGTAA-3' and 5'-CAAAGCCAGACAAAATGGCCC-3'), m*Ren* Tg (5'-CATCCACCGGATCTAGATAAC-3' and 5'-CAAAGCCAGACAAAATGGCCC-3'), h*REN* Tg (5'-GCTTTCTCAGCCAGGACATC-3' and 5'-TGCCAATGGCCTGTTCAATG-3'), and mouse *Gapdh* gene (5'-AAAATGGTGAAGGTCGGTGTG-3' and 5'-TGAGGTCAATGAAGGGGTCGT-3').

### Measurement of blood pressure

Systolic blood pressure was measured by a programmable sphygmomanometer (BP-98A; Softron, Tokyo, Japan) using the tail-cuff method, as previously described [2].

### Statistical analyses

Values are expressed as mean±SD. Number of animals analyzed is shown in each panel. All data were analyzed using an unpaired t-test. Results with *P*<0.05 were considered statistically significant.

## Results

### Expression of a 15-kb h*REN* Tg in THM

We first tested whether *renin* gene expression in THM appropriately regulated the maintenance of blood pressure homeostasis. To this end, we crossed female homozygous h*REN* TgM with male heterozygous h*AGT* TgM and obtained progeny carrying either h*REN* Tg alone (control; ctrl) or both h*REN* and h*AGT* Tgs (THM, Fig 1B). Total RNA was prepared from the kidneys and expression of the h*REN* Tg as well as the endogenous m*Ren* gene were analyzed by qRT-PCR. As anticipated, endogenous m*Ren* gene expression was significantly lower (female, 2.5-fold; male, 1.7-fold) in THM than in control mice (Fig 1C). In the same hypertensive environment, however, h*REN* Tg expression was upregulated (female, 4.2-fold; male, 6.0-fold; Fig 1D). These results indicated that the h*REN* Tg, but not the endogenous m*Ren* gene, was inappropriately regulated in THM from the perspective of blood pressure homeostasis.

### Generation of TgM carrying a m*Ren* gene fragment that is homologous to the 15-kb h*REN* Tg

Two possible explanations for dysregulation of the h*REN* Tg in THM seemed most likely. First, species-specific differences in their transcriptional regulatory mechanisms may account for the divergent phenotypes such that the regulatory elements contained in the h*REN* Tg are not able to function properly in the mouse environment. Second, the 15-kb h*REN* Tg fragment may lack a putative *cis*-element(s) that is required for its proper regulation in THM. In possible accord with the latter hypothesis, it has been reported that a 140-kb h*REN* P1 artificial chromosome Tg was appropriately downregulated in a hypertensive environment [17].

**Fig 2:**
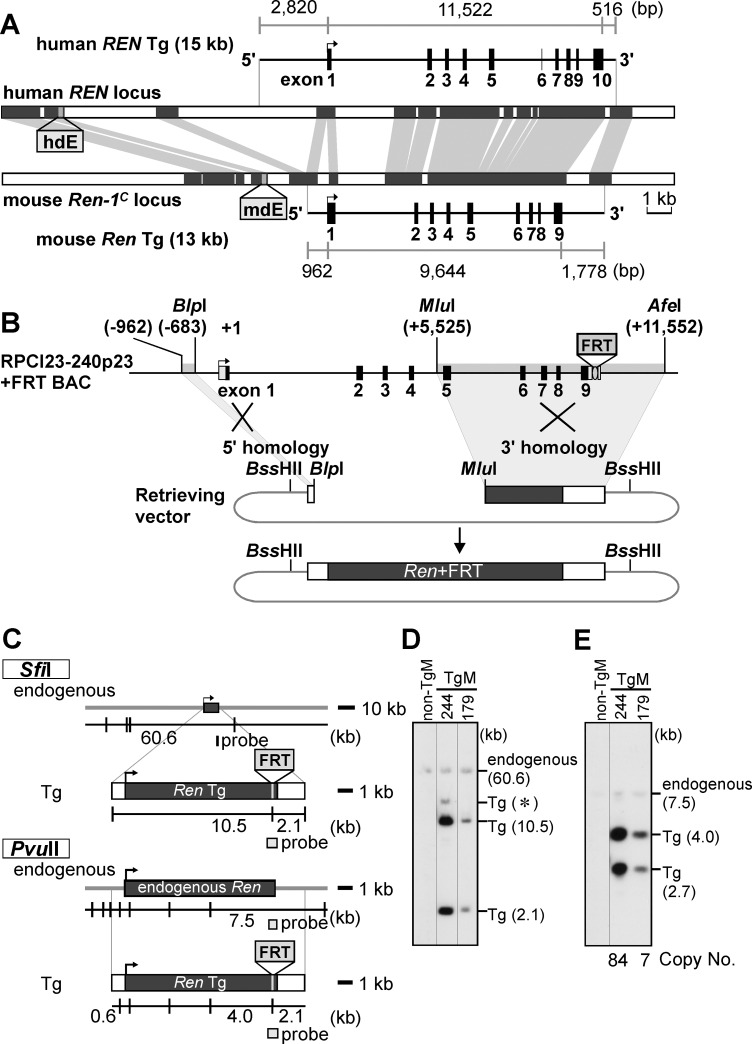
Generation of mouse *renin* transgenic mice. **(A)** Homology comparison of the human and mouse *Ren* gene loci determined by the Ensembl genome browser (http://www.ensembl.org, [29]). Conserved regions are indicated by shaded lines. Human *RENIN* (top, 15 kb) and mouse *renin* (bottom, 13 kb) Tg fragments used for microinjection are shown with their exon–intron organization. Lengths of the gene body, and the 5' and 3' flanking regions are shown in bp. (**B**) The RPCI23-240p23+FRT BAC carries the mouse *Ren* gene locus with the FRT sequences inserted at the 3'-untranslated region of the gene (top, [14]). Restriction enzyme sites with their positions relative to the transcriptional start site (+1) are shown. The 5' (nucleotides −962 to −683 relative to the transcription start site) and 3' (nt +5,525 to +11,552) homology fragments were prepared by PCR amplification and restriction enzyme digestion (*Mlu*I/*Afe*I), respectively, of the BAC clone and subcloned into the retrieving vector (middle). Following the retrieving reaction (bottom), the *Bss*HII fragment was released and used for microinjection. Gene body and flanking regions are shown as solid and open rectangles, respectively. (**C**) Partial restriction enzyme map of the mouse endogenous and Tg *Ren* gene loci. Knock-in of the FRT sequences (gray rectangle) generated artificial *Sfi*I and *Pvu*II sites in the Tg locus, which were used to discriminate the endogenous and Tg loci. The positions of restriction enzyme sites and expected restriction enzyme fragments with their sizes are shown beneath each map (top *Sfi*I, bottom *Pvu*II). The probe used for Southern blot analysis in D and E is indicated by a gray rectangle. (**D** and **E**) DNAs from thymic cells of non-Tg and Tg animals were digested with *Sfi*I (**D**) or *Pvu*II (**E**) in agarose plugs, separated by electrophoresis, and hybridized to the probe shown in C. On the right of each panel are expected bands with their sizes (in kb). A partial digestion product is marked by an asterisk. Signal intensities of the bands in (**E**) were quantified by phosphorimager, and the Tg copy numbers were estimated by calculating Tg/endogenous. ratios (beneath the panel). Tg, transgene. Lines in D and E indicate that lanes were run on the same gel but were noncontiguous.

To ask where the mouse ortholog of this putative human transcriptional regulatory sequence is located, as well as whether the h*REN* and m*Ren* genes share common regulatory mechanisms in maintaining blood pressure homeostasis, we generated TgM carrying a m*Ren* gene fragment that was homologous to the 15-kb h*REN* Tg. The homologous sequence was determined using the Ensembl genome browser (http://www.ensembl.org, [29]) and defined as a 13-kb fragment containing approximately 1 kb and 1.8 kb of 5'- and 3'-flanking sequences, respectively, of the m*Ren* gene (Fig 2A, bottom). The DNA sequences were retrieved from a modified BAC (RPCI23-240p23+FRT [14]) by defective prophage λ-Red recombineering (Fig 2B [27]). In this modified BAC, FRT sequences (133 bp with artificial *Sfi*I and *Pvu*II recognition sites) were inserted into the 3'-untranslated region of the mouse *Ren-1c* gene, which allows distinction between the endogenous and transgenic m*Ren* genes (Fig 2C). The 13-kb DNA fragment, released from the retrieving vector by *Bss*HII digestion, was injected into the pronuclei of fertilized mouse eggs. Tail DNA from offspring was analyzed by PCR and Southern blotting to screen for transgenesis (data not shown), and two TgM lines (244 and 179) were established. The integrity of the Tg was confirmed by the long-range structural analysis of high-molecular-weight thymic DNA (Fig 2D). Quantification of the signal intensities of Southern blot bands was used to estimate that the Tg copy numbers of lines 244 and 179 were 84 and 7, respectively (Fig 2E).

### Basal level Tg expression in the kidney of 13-kb m*Ren* TgM

**Fig 3:**
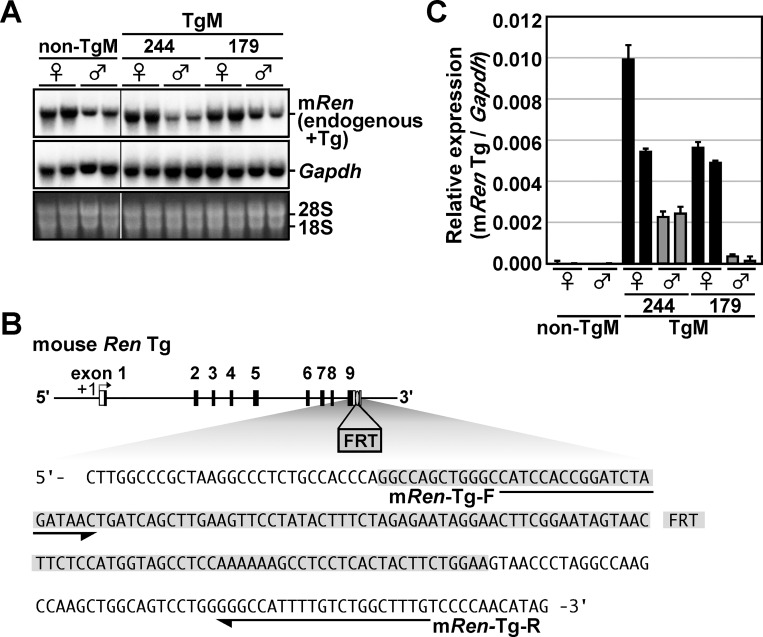
*Ren* gene expression in TgM. (**A**) Northern blot analysis of the TgM. Total RNA samples (20 μg) from the kidney of non-Tg and Tg animals (8-week old) were electrophoresed on a 1.2% agarose gel and subjected to Northern blot analysis with the mouse *Ren* (top) and *Gapdh* (middle) probes. The *Kpn*I/*Nco*I fragment (exons 3–9) from the mouse *Ren-1*^*C*^ cDNA was used for simultaneous expression analysis of the endogenous and Tg *Ren* genes. Ethidium bromide staining of the gel is shown at the bottom (the positions of 28S and 18S rRNA are indicated). Lines indicate that lanes were run on the same gel but were noncontiguous. (**B**) Sequences of the mouse *Ren* Tg (*Ren*+FRT). Hatched region is the FRT sequence. Positions of primers used for qRT-PCR in C are underlined. (**C**) Expression levels of the mouse *Ren* Tg were analyzed by qRT-PCR. Each value represents the ratio of m*Ren* Tg expression to that of *Gapdh*. Each sample was analyzed at least three times, and mean ± SD are shown.

Multiple copies of the m*Ren* transgene in the TgM suggested that it could be overexpressed. Therefore, we conducted Northern blot analysis using m*Ren* cDNA probe to roughly quantify the overall (endogenous plus transgenic) m*Ren* gene expression (Fig 3A). Unexpectedly, m*Ren* mRNA levels did not differ significantly between non-TgM and TgM lines. To precisely quantify the expression levels of the Tg, we performed qRT-PCR analysis. Tg-specific primers were designed based on the FRT sequences present only in the 3'-untranslated region of the m*Ren* Tg (Fig 3B). The results in Fig 3C revealed that the Tg was in fact expressed, but as shown in Fig 3A, the levels were much lower than those of the endogenous gene. This result was rather beneficial for our studies because blood pressure elevation due to overexpression of m*Ren* Tg alone in the basal state (non-THM situation) was not suitable for the analysis of its regulation in hypertensive environment. We then measured the systolic blood pressure of non-Tg and Tg animals and found their levels did not differ significantly, as expected from the Tg expression level (Table 1). Therefore, we decided to use these 13-kb m*Ren* TgM as reporter lines in gene expression analyses *in vivo*.

**Table 1:** Comparison of systolic blood pressure (SBP) between non-TgM and m*Ren* TgM.

	Female			Male		
	non-TgM	m*Ren* TgM		non-TgM	m*Ren* TgM	
		line 244	line 179		line 244	line 179
n	6	4	4	7	3	4
SBP (mmHg)	109.5 ± 4.3	114.3 ± 5.5	115.4 ± 16.7	109.2 ± 5.5	104.3 ± 2.6	106.0 ± 10.8

n, total number of mice analyzed in each group.

### 13-kb m*Ren* Tg expression in the kidneys of THM

**Fig 4:**
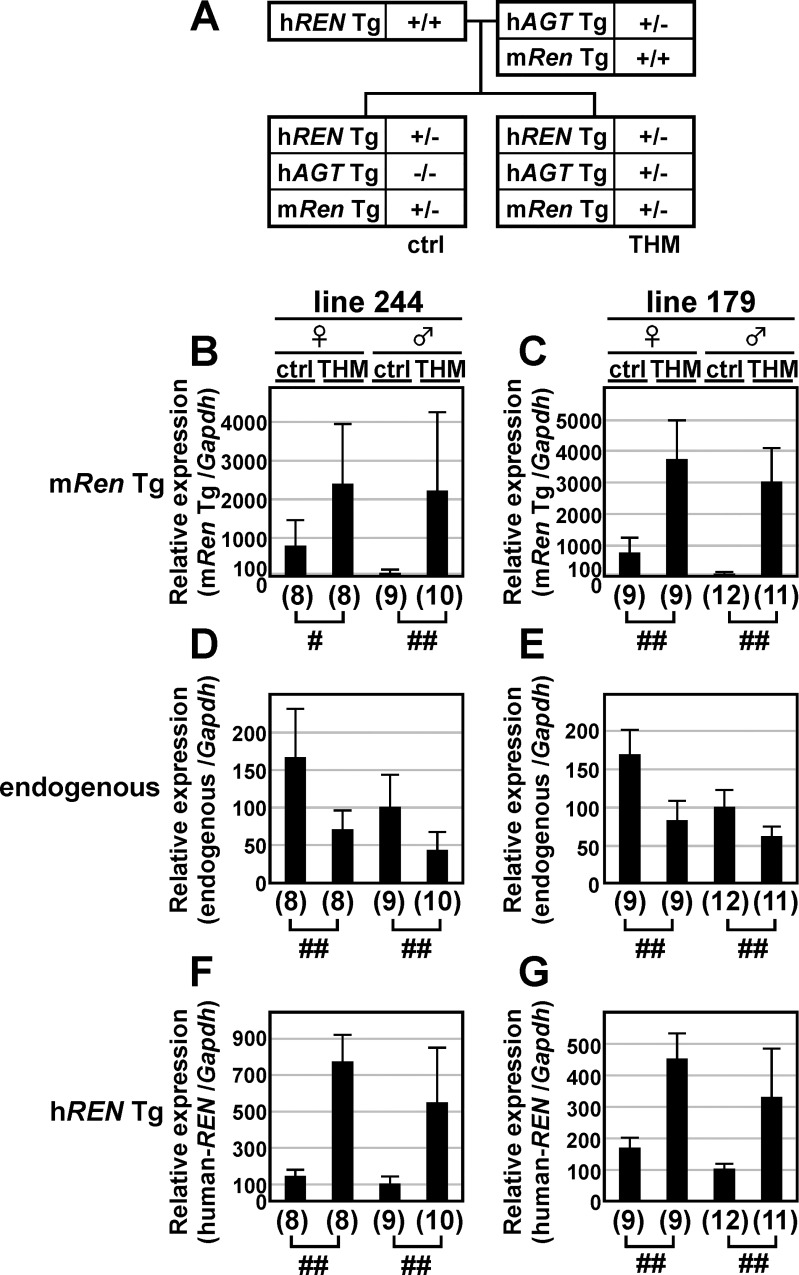
Expression of the mouse *Ren* Tg in THM. (**A**) Breeding strategy for introducing mouse *Ren* Tg (m*Ren* Tg) into normotensive (ctrl) or hypertensive (THM) mice. (**B**-**G**) Total RNA was isolated from the kidney of normotensive (ctrl) or hypertensive (THM) animals (8-week old) and subjected to qRT-PCR analyses. Expression levels of mouse *Ren* Tg (m*Ren* Tg; lines 244 and 179 in B and C, respectively), endogenous *Ren* (endogenous, D and E), human *REN* Tg (h*REN* Tg; F and G), and *Gapdh* (data not shown) were determined. Each value represents the ratio of m*Ren* Tg, endogenous, or h*REN* Tg expression to that of *Gapdh*. Expression value of male control animals in each group was arbitrarily set at 100. qPCR analyses were repeated twice. Number of animals analyzed is shown in parentheses below each panel and mean ± SD are shown. Statistically significant differences between ctrl and THM were determined using an unpaired t-test (#, *P* < 0.05; ##, *P* < 0.01).

To examine its regulation in THM, the 13-kb m*Ren* Tg allele was bred onto a normotensive or hypertensive (THM) environment (Fig 4A). Total RNA was extracted from the kidneys of the mice, and the abundance of m*Ren* Tg (Fig 4B and 4C), endogenous m*Ren* (Fig 4D and 4E), and h*REN* Tg (Fig 4F and 4G) transcripts was determined by qRT-PCR. In the THM, while endogenous m*Ren* gene expression was suppressed, m*Ren* Tg (female, 3.1~5.1-fold; male, 22.2~30.3-fold) and h*REN* Tg (female, 2.6~5.6-fold; male, 3.5~5.5-fold) expression was significantly upregulated in the same RNA samples. These results suggest that the dysregulation of h*REN* Tg in THM was not due to species-specific differences in transcriptional regulation, but rather to the lack of putative silencing elements in both the 13-kb m*Ren* and 15-kb h*REN* Tgs.

### Assessment of a possible role for the mdE enhancer in the kidney of THM

**Fig 5:**
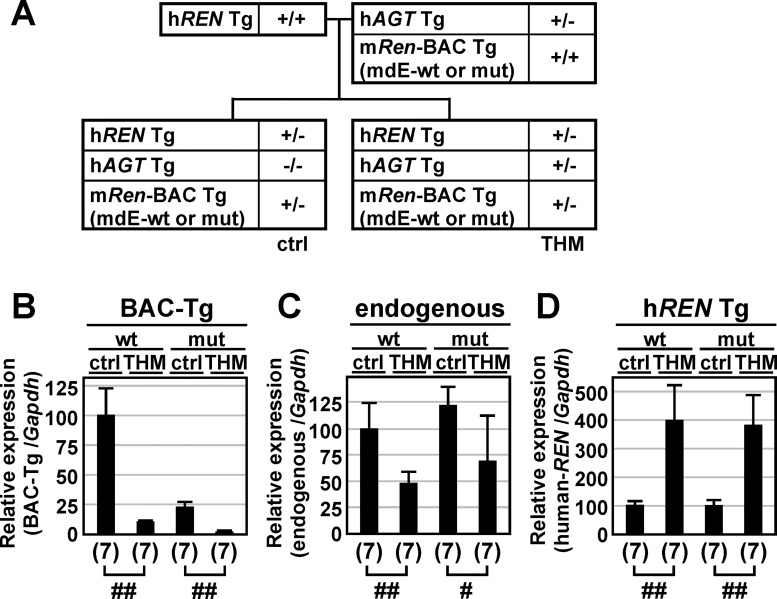
Expression of the mouse *Ren* BAC Tg in THM. (**A**) Breeding strategy for introducing the mouse *Ren* BAC Tg (mdE wild-type, wt, or mutant, mut) into normotensive (ctrl) or hypertensive (THM) mice. (**B**–**D**) Total RNA was isolated from the kidney of normotensive or hypertensive animals (8-week old) and subjected to qRT-PCR analyses. Expression levels of mouse *Ren* BAC Tg (BAC-Tg; B), endogenous *Ren* (endogenous; C), human *REN* Tg (h*REN* Tg; D), and *Gapdh* (data not shown) were determined. Each value represents the ratio of BAC-Tg, endogenous, or h*REN* Tg expression to that of *Gapdh*. Expression value of wt control animals in each group was arbitrarily set at 100. Number of animals analyzed is shown in parentheses below each panel and mean ± SD are shown. Statistically significant differences between ctrl and THM were determined using an unpaired t-test (#, *P* < 0.05; ##, *P* < 0.01).

Because a putative silencing element of the m*Ren* gene in THM was inferred to be located outside of the 13-kb gene fragment that was used for transgenic analysis, we focused on mdE, an enhancer sequence that resides approximately 3 kb upstream of the m*Ren* transcription start site and is not contained in the 13-kb m*Ren* fragment (Fig 2A). Its human ortholog is present approximately 12 kb upstream of the h*REN* transcriptional start site, and the 15-kb h*REN* Tg fragment did not bear this sequence (Fig 2A). To assess the role of mdE in the kidneys of THM, we employed previously generated 156-kb m*Ren* BAC TgM lines [22]. This set of TgM lines carry either wild-type (wt) or mdE-null (mut) m*Ren* BACs, both integrated at the identical chromosomal site. These alleles were subjected to a normotensive or hypertensive (THM) environment, as was the case for the 13-kb m*Ren* Tg (Fig 4A), and their expression levels were determined by qRT-PCR analysis of total kidney mRNAs (Fig 5A). As shown in Fig 5B, the expression of mdE-null (mut) m*Ren* Tg was significantly lower than that of mdE-wt m*Ren* Tg when analyzed in the normotensive environment, which was consistent with our previous results [22]. In the hypertensive environment (THM), the expression of the mdE-wt m*Ren* Tg was significantly suppressed, demonstrating that 156-kb m*Ren* sequences carried sufficient information to confer appropriate regulation. The expression of the mdE-mut m*Ren* Tg was further suppressed in the hypertensive environment (THM), demonstrating that the mdE sequence is dispensable for the observed transcriptional downregulation in the THM. Endogenous m*Ren* (Fig 5C) and h*REN* Tg (Fig 5D) expression levels in these RNA samples were similar to the previous results (Figs 1 and 4). These results revealed that the 156-kb m*Ren* fragment possesses sequences conferring proper regulatory information in THM, and that mdE is dispensable for this function.

### Generation of 5'-large-deletion mutant and FRT-knock-in pseudo WT alleles of endogenous m*Ren* gene by genome editing

**Fig 6:**
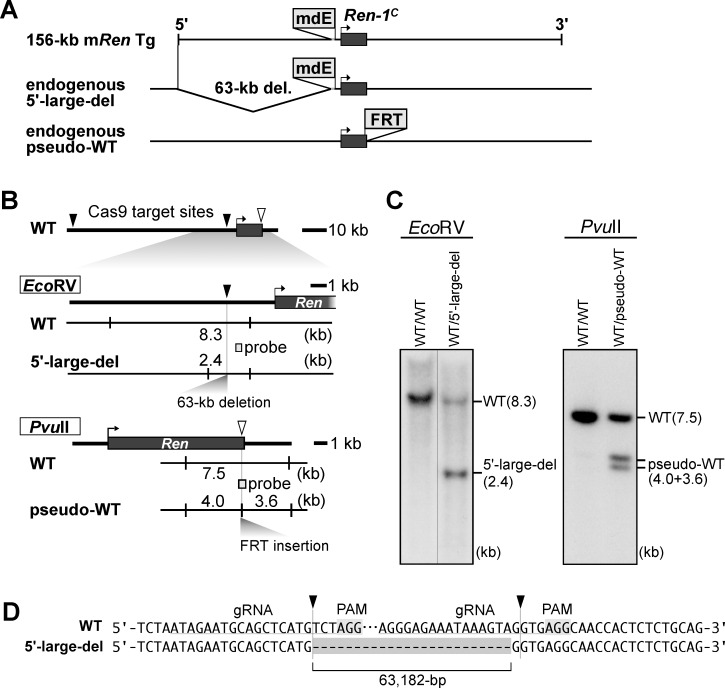
Generation of endogenous mouse renin mutant alleles by genome editing. **(A)** Schematic representation of the 156-kb m*Ren* Tg, endogenous 5'-large-del and endogenous pseudo-WT alleles. Positions of mdE and FRT sequences are indicated by gray rectangles. **(B)** Partial restriction enzyme maps of the mouse endogenous WT and mutant alleles. The Cas9 target sites for generating 5'-large-del and pseudo-WT alleles are shown by solid and open arrowheads, respectively. Targeting at two upstream sites removes 63-kb sequence from the 5'-upstream region of m*Ren* gene, generating a 2.4-kb *Eco*RV restriction fragment in the mutant allele. Knock-in of the FRT sequence at a downstream site introduces artificial *Pvu*II site, generating 4.0- and 3.6-kb *Pvu*II restriction fragments in the mutant allele. Probes used for Southern blot analysis in C are indicated by gray rectangles. **(C)** DNAs of WT and mutant animals were digested with *Eco*RV or *Pvu*II, separated by electrophoresis, and hybridized to the probes shown in B. Shown on the right of each panel are expected bands with their sizes (in kb). **(D)** Sequence alignment of WT (reference) and 5'-large-del alleles confirmed the 63-kb sequence deletion in the 5'-large-del allele of m*Ren* gene. PAM and g(uide)RNA sequences are shaded and underlined, respectively. Cleavage sites predicted from PAM locations are indicated by arrowheads.

The previous results implied that putative silencing element(s) resided within the 156-kb region of the locus examined, yet outside of the 13-kb region of the m*Ren* gene. We therefore decided to delete the 5'-upstream region of the endogenous m*Ren* gene (63-kb; 5'-large-del; Fig 6A) using CRISPR/Cas9 genome editing [30]. Guide RNAs were designed to target 5'-ends of the 156-kb BAC Tg and of the mdE to remove intervening sequence between these sites (Fig 6B). Following microinjection of two targeting plasmids into pronuclei, correct recombination events in the founder mice were confirmed by Southern blot and sequencing analyses (Fig 6B–6D). Aside from this mutation, the 3'-untranslated region of the endogenous, wild-type m*Ren* allele was marked by FRT insertion to generate a pseudo-WT allele (Fig 6A, bottom), to discriminate its expression from that of the 5'-large-deletion allele in the same sample. CRISPR/Cas9-mediated genome editing was used for the mutagenesis, and correct recombination was confirmed by Southern blot and DNA sequencing analyses (Fig 6B and 6C and data not shown).

### 5'-large-deletion m*Ren* gene expression in the kidneys of THM

**Fig 7:**
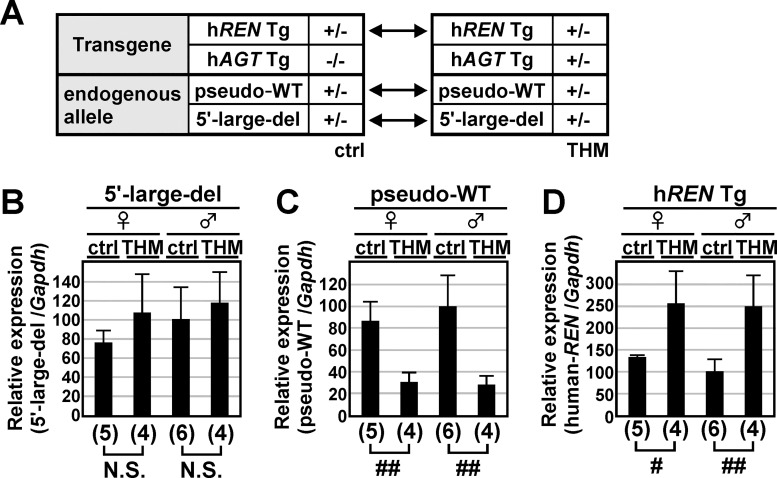
m*Ren* gene expression in the 5'-large-del mutant allele. (**A**) Expression of m*Ren* gene in the 5'-large-del mutant allele was analyzed in normotensive (ctrl) and hypertensive (THM) mouse environments. (**B**–**D**) Total RNA was isolated from the kidney of normotensive or hypertensive animals (8-week old) and subjected to qRT-PCR analyses. Expression levels of endogenous 5'-large-del m*REN* (B), endogenous pseudo-WT m*REN* (C), h*REN* Tg (D), and *Gapdh* (data not shown) were determined. Each value represents the ratio of renin genes expression to that of *Gapdh* and mean ± SD is shown. Values of male control animals in each group was arbitrarily set at 100. Number of animals analyzed is shown in parentheses below each panel. Statistically significant differences between ctrl and THM were determined using an unpaired t-test (N.S., not significant; #, *P* < 0.05; ##, *P* < 0.01).

5'-large-del allele in combination with pseudo-WT allele was bred onto a normotensive or hypertensive (THM) environment (Fig 7A). Total RNA was extracted from the kidneys of the mice, and the abundance of endogenous 5'-large-del m*Ren* (Fig 7B), endogenous pseudo-WT m*Ren* (Fig 7C), and h*REN* Tg (Fig 7D) transcripts was determined by qRT-PCR. Expression of pseudo-WT m*Ren* gene was suppressed in THM (Fig 7C), as was seen in the true wild-type allele (Figs 1C, 4D, 4E and 5C), while that of h*REN* Tg was upregulated in THM (Fig 7D). In the same set of samples, however, expression of 5'-large-del m*Ren* gene in THM did not differ significantly from that of the control animals (Fig 7B). These results clearly demonstrated that putative silencing element(s) in THM reside within the 63-kb upstream region of the m*Ren* gene that was deleted in the 5'-large-del allele.

### 13-kb m*Ren* Tg expression in the fetal and neonatal kidneys of THM

**Fig 8:**
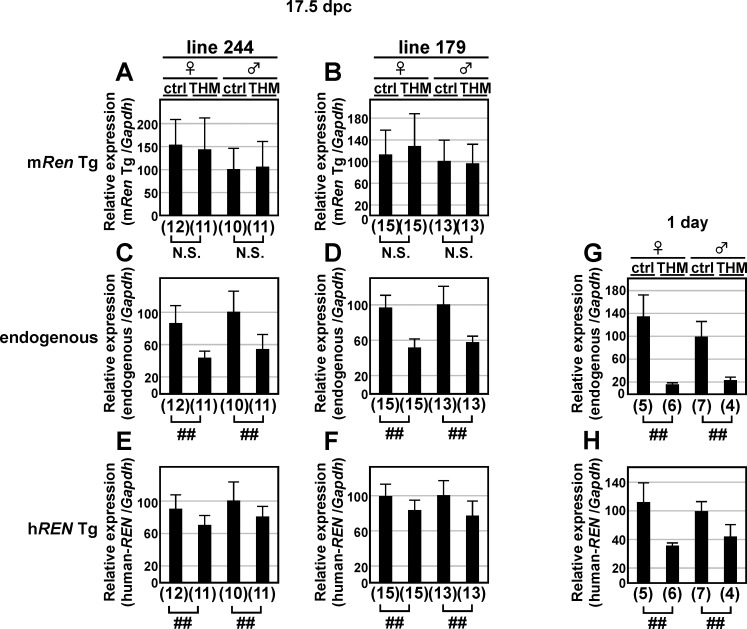
*Renin* genes expression in the fetal and neonatal kidney of THM. (**A**–**F**) Total RNA was isolated from the kidney of 17.5-dpc m*Ren* TgM carrying either h*REN* Tg alone (control) or both h*REN* and h*AGT* genes (THM) and subjected to qRT-PCR analyses. Expression levels of mouse *Ren* Tg (m*Ren* Tg; lines 244 and 179 in A and B, respectively), endogenous *Ren* (endogenous; C and D), human *REN* Tg (h*REN*; E and F), and *Gapdh* (data not shown) were determined. (**G**–**H**) Total RNA was isolated from the kidney of neonatal (one-day-old) TgM carrying either h*REN* Tg alone (control) or both h*REN* and h*AGT* genes (THM) and subjected to qRT-PCR analyses. Expression levels of endogenous m*Ren* gene (endogenous; G), human *REN* Tg (h*REN*; H), and *Gapdh* (data not shown) were determined. Each value represents the ratio of endogenous or h*REN* Tg expression to that of *Gapdh*. Expression value of male control animals in each group was arbitrarily set at 100. qPCR analyses were repeated twice. Number of animals analyzed is shown in parentheses below each panel and mean ± SD are shown. Statistically significant differences between ctrl and THM were determined using an unpaired t-test (N.S., not significant; ##, *P* < 0.01).

We previously reported that mating of male h*REN* TgM (15 kb) and female h*AGT* TgM produced a severe hypertension phenotype during pregnancy of the mother (pregnancy-associated hypertension, PAH). It was therefore predicted that fetoplacentally-produced hREN enters the maternal circulation where it catalyzes maternally produced hAGT to cause blood pressure elevation [31]. We therefore tested the hypothesis that the lack of a silencing element led to dysregulation of the 15-kb h*REN* Tg expression in the fetus by examining *renin* gene expression in the fetal kidney of THM. To obtain control and THM fetuses in the same litter, we set up the mating as shown in Fig 4A. Kidneys were collected from 17.5 days post-coitum (dpc) fetuses, and *renin* gene expression was analyzed by qRT-PCR. Expression of the endogenous m*Ren* gene was suppressed in THM when compared with that in the control littermates (Fig 8C and 8D). Although the expression of both 13-kb m*Ren* and h*REN* Tgs was upregulated in the kidneys of adult THM (Fig 4B, 4C, 4F and 4G), m*Ren* Tg expression did not differ significantly between THM and control animals (Fig 8A and 8B), and h*REN* Tg expression was even downregulated appropriately in THM (Fig 8E and 8F).

We then collected kidneys from newborn (1-day-old) THM, analyzed *renin* gene expression by qRT-PCR (Fig 8G and 8H), and found that expression of both endogenous m*Ren* and h*REN* Tg was appropriately suppressed in THM. These results demonstrated that dysregulation of the short *renin* Tgs in THM commenced sometime within the two months after birth.

## Discussion

Thanks to recent advances in locus-wide (3C, [32]) and even genome-wide (Hi-C, [33]) methodologies for identifying interactions between distinct genomic DNA segments, a number of long-range interactions between promoter and distal regulatory elements have been reported [34, 35]. Although it has been implied that the hypothetical silencing element is located far away from its protein-coding region of the h*REN* gene [17], equivalent findings in the mouse have not been documented. In this study, we showed that a putative silencing element of the m*Ren* gene was also distally located to its protein-coding region, suggesting that transcriptional mechanisms, at least in adult kidney, are conserved between the two species (Table 2).

**Table 2:** Expression of human and mouse *renin* Tgs in adult and fetal THM.

		*Renin* gene expression in h*REN*/h*AGT* double TgM (THM)		
		Adult	Fetus	
13-kb Tg	human	↑	N.D.	Thompson *et al*.^3^
15-kb Tg	human	↑	↓	This study
140-kb Tg	human	↓	N.D.	Sinn *et al*.^17^
13-kb Tg	mouse	↑	→	This study
156-kb Tg	mouse	↓	N.D.	This study
endo. wild-type	mouse	↓	↓	This study
endo. 5'-large-del	mouse	→	N.D.	This study

Tg, transgene; endo., endogenous; ↓, suppressed; ↑, induced; →, not changed; N.D., not determined.

Both the human distal enhancer (hdE) and mdE are distally located to the *renin* genes [18, 19] and bear multiple transcription factor binding sites including a cAMP-responsive element (CRE). Desch *et al*. reported that CREs in hdE and proximal promoter region of the h*REN* gene were essential for its correct transcriptional regulation in response to administration of β-adrenergic receptor agonist and low sodium diet in TgM [36]. Although we have discovered an indispensable role of mdE in basal m*Ren* gene transcription [22], its possible role in the hypertensive environment has not been tested. Therefore, they were possible candidates for *cis*-elements responsible for transcriptional repression of *renin* genes in THM (Fig 2A). Previous [37] and our current results (Fig 5) demonstrated that both hdE and mdE are dispensable for transcriptional suppression of *renin* genes in THM. In other words, novel distal elements confer appropriate regulation of the h*REN* and m*Ren* genes in THM. This notion was further supported by the fact that deleting the 63-kb upstream region of the endogenous m*Ren* gene abrogated its transcriptional repression in THM. This result not only demonstrated the existence of a putative silencing element within the m*Ren* gene locus, but also ensured that we could ultimately identify the element by CRISPR/Cas9-mediated mutagenesis of the locus. It must be noted that upregulation of the 5'-large-del m*Ren* gene was not observed in THM, which was the case for short *renin* Tgs. Deductively, it was suggested that additional *cis*-element, possibly located 3' to the gene, may be suppressing such an aberrant activation. In addition, what kind of physiological stimuli in THM (e.g. pressure overload, high concentration of AII) is in fact modulating *renin* gene transcription in THM remains to be determined.

We inferred that aberrant regulation of the 15-kb h*REN* Tg is the basis for pathogenesis of hypertension in adult THM. Furthermore, we hypothesized that the same dysregulation may be associated with the expression of short *renin* genes in the fetal kidney, which may be, at least in part, a cause of the maternal hypertension in PAH model animals [31]. To test this hypothesis, we analyzed the kidney RNA of fetuses with THM genotype (17.5 dpc) and found that the expression of neither h*REN* (15 kb) nor m*Ren* (13 kb) Tgs were upregulated (Fig 8A, 8B, 8E and 8F, Table 2). The expression of h*REN* Tg was rather downregulated appropriately in THM (Fig 8E and 8F, Table 2), as was the case for endogenous m*Ren* gene (Fig 8C and 8D). As far as we know, method to measure fetal blood pressure in mice has not been developed. However, because of abundant expression of h*AGT* Tg in the liver of fetal mouse (17.5-dpc, [38]) and reduced endogenous m*Ren* gene transcription in fetuses (Fig 8C & 8D), we assume hypertensive condition and/or high concentration of plasma angiotensin II exists in the mouse fetus with THM genotype. In support of this notion, it has been reported that direct infusion of angiotensin II into a fetal lamb raised its blood pressure and suppressed plasma renin activity [39]. Therefore, it is predictable that 15-kb h*REN* Tg expression in the fetal kidney (Fig 8E and 8F) was suppressed by increased angiotensin II production or hypertensive stimulus.

We previously reported an overexpression of the 15-kb h*REN* Tg in the placenta of THM during late gestation [31], while it was reported that endogenous h*REN* mRNA levels are higher during early gestation than those during term in human placenta [40]. It is therefore possible that a lack of distal regulatory elements in the 15-kb h*REN* Tg caused its aberrant expression in the placenta and pathogenesis of PAH. Germain *et al*. identified a *cis*-regulatory element between −5.8 and −5.5 kb upstream from the transcription start site of h*REN* in primary human chorionic cells [41]. A lack of such an element in the 15-kb h*REN* Tg may account for the dysregulation.

In the same way as in THM fetuses, h*REN* Tg expression was appropriately suppressed in the kidney of THM neonates (1-day-old), demonstrating that regulation of short *renin* Tg expression in THM changed during two months period after birth. Alteration in transcriptional environment of the kidney or epigenetic change in proximal promoter region of *renin* genes by long-term exposure to high concentration of AII and/or high blood pressure, in combination with a lack of distal sequences may account for the phenotype. Although exact reason for developmental difference of the *renin* gene regulation in THM remains to be determined, it is certain that distal silencing element is essential for proper regulation of *renin* genes in the adult THM, because expression of the endogenous m*Ren* gene in THM was properly regulated throughout life.

In summary, our results demonstrate that putative silencing elements of both h*REN* and m*Ren* genes in the kidney of adult THM are located at long distance from their protein-coding regions. In the m*Ren* gene, mdE was dispensable for the repression in THM and approximate location of a putative silencing element was determined, by genome editing, to be upstream of mdE and inside a 156-kb m*Ren* gene locus. Meanwhile, transcriptional responses of the short *renin* transgenes in the fetal/neonatal vs adult kidneys were apparently different, although endogenous m*Ren* gene behaved similarly at all these stages. We therefore conclude that distal silencing element of the m*Ren* gene (and probably that of h*REN* gene, too) was essential for proper regulation of *renin* genes in adult THM.

## Abbreviations

ACE: angiotensin-converting enzyme
AGT: angiotensinogen
BAC: bacterial artificial chromosome
CRE: cyclic AMP-response element
dpc: days post coitum
FRT: flip recombinase recognition target
hdE: human distal enhancer
h*REN*: human *RENIN*
JG: juxtaglomerular
mdE: mouse distal enhancer
m*Ren*: mouse *Renin*
PAH: pregnancy-associated hypertension
Tg: transgene
TgM: transgenic mice
THM: Tsukuba hypertensive mice
wt: wild-type
